# Human FcRn Is a Two-in-One Attachment-Uncoating Receptor for Echovirus 18

**DOI:** 10.1128/mbio.01166-22

**Published:** 2022-07-05

**Authors:** Xiangpeng Chen, Xiao Qu, Congcong Liu, Yong Zhang, Guigen Zhang, Pu Han, Yali Duan, Qi Li, Liang Wang, Wenjing Ruan, Peiyi Wang, Wensheng Wei, George F. Gao, Xin Zhao, Zhengde Xie

**Affiliations:** a Beijing Key Laboratory of Pediatric Respiratory Infection Diseases, Key Laboratory of Major Diseases in Children, Ministry of Education, National Clinical Research Center for Respiratory Diseases, Research Unit of Critical Infection in Children, Chinese Academy of Medical Sciences, 2019RU016, Laboratory of Infection and Virology, Beijing Pediatric Research Institute, Beijing Children’s Hospital, Capital Medical University, National Center for Children’s Health, Beijing, China; b CAS Key Laboratory of Pathogen Microbiology and Immunology, Institute of Microbiology, Chinese Academy of Sciences, Beijing, China; c Institute for Hepatology, National Clinical Research Center for Infectious Disease, Shenzhen Third People’s Hospital, Shenzhen, China; d Cryo-EM Centre, Department of Biology, Southern University of Science and Technology, Shenzhen, China; e National Laboratory for Poliomyelitis, WHO WPRO Regional Polio Reference Laboratory, National Health Commission Key Laboratory for Biosafety, National Health Commission Key Laboratory for Medical Virology, National Institute for Viral Disease Control and Prevention, Chinese Center for Disease Control and Prevention, Beijing, China; f Center for Biosafety Mega-Science, Chinese Academy of Sciences, Wuhan, China; g Institute of Human Virology, Key Laboratory of Tropical Disease Control of Ministry of Education, Zhongshan School of Medicine, Sun Yat-sen University, Guangzhou, Guangdong, China; h Division of Life Sciences and Medicine, University of Science and Technology of China, Hefei, China; i Biomedical Pioneering Innovation Center, Beijing Advanced Innovation Center for Genomics, Peking-Tsinghua Center for Life Sciences, Peking University Genome Editing Research Center, State Key Laboratory of Protein and Plant Gene Research, School of Life Sciences, Peking University, Beijing, China; j CAS Center for Influenza Research and Early-Warning (CASCIRE), Chinese Academy of Sciences, Beijing, China; University of Calgary

**Keywords:** CRISPR/Cas9, FcRn, echovirus, enterovirus, receptors

## Abstract

Virus-receptor interactions determine viral host range and tissue tropism. CD55 and human neonatal Fc receptor (FcRn) were found to be the binding and uncoating receptors for some of the echovirus-related enterovirus species B serotypes in our previous study. Echovirus 18 (E18), as a member of enterovirus species B, is a significant causative agent of aseptic meningitis and viral encephalitis in children. However, it does not use CD55 as a critical host factor. We conducted CRISPR/Cas9 knockout screening to determine the receptors and entry mechanisms and identified FcRn working as a dual-function receptor for E18. Knockout of *FCGRT* and *B2M*, which encode the two subunits of FcRn, prevented infection by E18 and other echoviruses in the same physiological cluster. We then elucidated the underlying molecular mechanism of receptor recognition by E18 using cryogenic electron microscopy. The binding of the FCGRT subunit to the canyon region rotates the residues around the pocket, triggering the release of the pocket factor as observed for other enterovirus species B members.

## INTRODUCTION

Echoviruses belong to enterovirus species B (EV-B) of the *Enterovirus* genus within the *Picornaviridae* family, which are single-stranded, positive RNA viruses (~30 nm) without a lipid envelope (nonenveloped virus). The genome length is approximately 7,410 nucleotides with a single open reading frame encoding four structural and seven nonstructural proteins. EV-B is the most commonly detected species worldwide and is often detected in patients with central nervous system (CNS)-associated infections and in cerebrospinal fluid (CSF) samples (1, 2). An overview of the worldwide enterovirus (EV) prevalence and distribution showed that echovirus 30 (E30), E6, coxsackievirus B5, E9, and E18 were the five most common serotypes with the highest prevalence in EV-B (1). Echoviruses can cause a wide variety of clinical symptoms, including mild symptoms of rashes and hand-foot-and-mouth disease (HFMD) as well as serious diseases such as viral encephalitis (VE), aseptic meningitis (AM), hepatitis, neonatal sepsis, and acute flaccid paralysis (3–6). However, no approved vaccines or effective antiviral drugs are available to treat echovirus infections.

E18 is an important viral pathogen in children; it has been reported frequently in China and is common in other countries worldwide (7–14). E18 is one of the most common enterovirus serotypes in nonpolio enterovirus detection in the United States and is identified mainly in infants younger than 1 year old (5, 15–17). CSF was the most common source of E18 detection, and death occurred in 1.8% of the reported cases with known outcomes in the United States from 1975 to 2005 (5). In Europe, E18 was the sixth most common enterovirus serotype in nonpolio enterovirus detections from 2015 to 2017 (18). E18-associated CNS infections have also been reported frequently in children in China (19, 20). An outbreak of E18-associated AM in Taiwan in 2006 was reported, and E18 accounted for 27.4% of the total EV-positive cases (13). We previously reported an E18-associated outbreak of encephalitis/meningitis in children in Hebei Province in China (12, 21). In our analysis, E18 was the main pathogen associated with VE/AM in children in 2015, accounting for 74.4% of all enteroviruses detected in children with VE/AM CSF specimens.

CD55, also known as decay-accelerating factor, has been reported to be an attachment receptor for some EV-B serotypes (22, 23). Previous studies from our group and Morosky et al. reported that human neonatal Fc receptor (FcRn) is EV-B’s entry and uncoating receptor (24, 25). We also found that some EV-B serotypes, such as E6, use a dual-receptor system for entry. In this system, the virus binds to CD55 on the cell surface and accomplishes the uncoating process in the endosome with the help of FcRn (24). Despite E18 being a significant pathogen of VE/AM worldwide, few studies have focused on the molecular invasion mechanism of E18. Additionally, echoviruses in the same evolutionary branch as E18, E2, and E15 have rarely been reported, and no high-resolution structural information is available for E18 before and after binding to its receptor.

This study certified that FcRn is the primary receptor for E18. FcRn, but not CD55, is a functional receptor for E2, E15, and E18. These echovirus infections can be completely abolished in human cell lines after the knockout (KO) of one of the FcRn-encoding genes, *FCGRT*, and susceptibility was restored by complementation with *FCGRT*. CD55 does not act as a receptor for these three echovirus serotypes. We used high-resolution cryogenic electron microscopy (cryo-EM) structures to identify the interaction and uncoating mechanisms of FcRn and E18. Rotation of residues around the pocket caused by FcRn binding initiates pocket factor release. The release of the pocket factor was further accelerated when the pH was lowered.

## RESULTS

### FcRn is a functional receptor for infection by E18 and echovirus in the same cluster.

**FIG 1:**
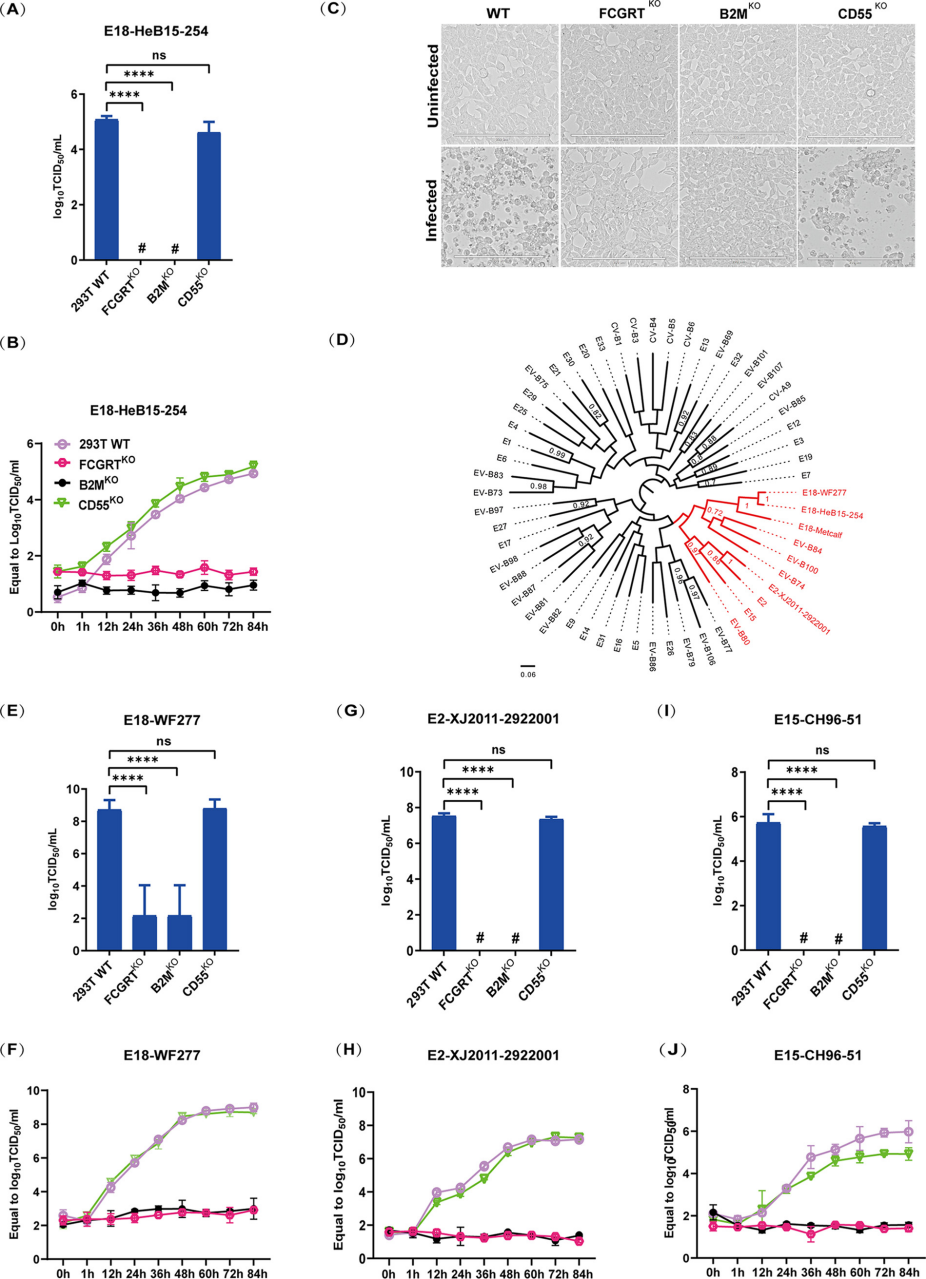
FcRn, but not CD55, is an essential receptor for E18, E2, and E15. (A, E, G, and I) Virus titers in the supernatants of HEK293T wild-type (293T WT), 293T *FCGRT*^KO^, 293T *B2M*^KO^, and 293T *CD5*5^KO^ cells infected with E18-HeB15-254 (A), E18-WF277 (E), E2 (G), and E15 (I). The TCID_50_ was determined in RD cells at 96 h postinfection. Data depict the means with standard errors of the means (SEM) for triplicates (*n* = 3). (B, F, H, and J) Growth curve analyses of E18-HeB15-254 (B), E18-WF277 (F), E2 (H), and E15 (J) in 293T WT, *FCGRT*^KO^, *B2M*^KO^, and *CD55*^KO^ cells. Data depict the means with SEM for triplicates (*n* = 3). (C) Light microscopy images of 293T cell lines infected with E18. 293T WT, 293T *FCGRT*^KO^, 293T *B2M*^KO^, and 293T *CD55*^KO^ cells were used. (D) Phylogenetic analysis of enterovirus species B based on *VP1* gene sequences. Significance was evaluated by using an unpaired two-tailed *t* test with Welch’s *post hoc* correction. #, undetected. ****, *P < *0.0001; ns, not significant.

To determine whether the previously identified dual-receptor system of CD55 and FcRn for enterovirus species B is the receptor for E18, *FCGRT*^KO^ (*FCGRT* knockout), *B2M*^KO^, and *CD55*^KO^ cell lines were used for E18 infection (24). No obvious viral replication was observed in either *FCGRT*^KO^ or *B2M*^KO^ HEK293T (293T) cells (Fig. 1A and B). No cytopathic effect (CPE) was detected in E18-infected *FCGRT*^KO^ or *B2M*^KO^ 293T cells, whereas E18 infection-induced CPE was detected in *CD55*^KO^ 293T cells and wild-type (WT) 293T cells (Fig. 1C).

A phylogenetic tree based on the *VP1* gene sequences of all EV-B serotypes showed that E2, E15, EV-B74, EV-B80, EV-B84, and EV-B100 clustered together with E18 (Fig. 1D). To further analyze whether FcRn and CD55 also served as the essential receptors for the echoviruses in this cluster, we tested another E18 strain (E18-WF277), E2, and E15. *FCGRT*^KO^ and *B2M*^KO^ abolished viral infection by E18, E2, and E15, suggesting that FcRn is a key host factor for these viruses (Fig. 1E to J). Previous studies have shown that CD55 acts as an attachment receptor on the cell surface (24). However, in contrast to *FCGRT*^KO^ and *B2M*^KO^ cells, *CD55*^KO^ did not affect infection by the two E18 strains in this study (Fig. 1A to C, E, and F). We further identified that *CD55*^KO^ could not prevent E2 or E15 infection (Fig. 1G to J).

### FcRn functions as a two-in-one receptor for E18 infection.

**FIG 2:**
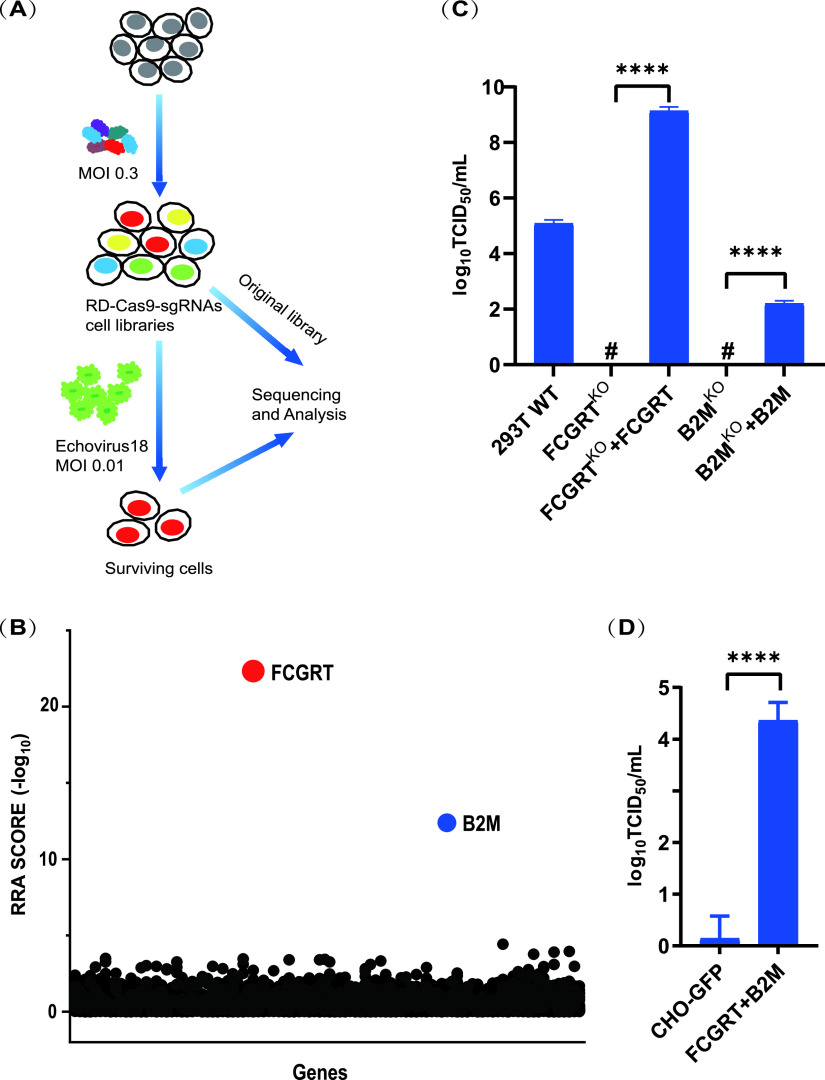
FcRn is a fundamental receptor for E18 infection. (A) Schematic of E18 receptor CRISPR/Cas9 screening in RD cells. (B) Enrichment of gene expression after E18 infection versus an uninfected control. Genes were rank ordered according to the robust rank aggregation (RRA) scores calculated by MAGeCK. (C) Viral titers in the supernatants of cell lines infected with E18. 293T WT cells, 293T *FCGRT*^KO^ cells, 293T *B2M*^KO^ cells, 293T *FCGRT*^KO^ cells complemented with lentivirus expressing FCGRT, and 293T *B2M*^KO^ cells complemented with lentivirus expressing B2M were used. (D) Viral titers in the supernatants of wild-type and FCGRT- and B2M-coexpressing CHO cell lines. TCID_50_ data were calculated by the Reed-Muench method. Experiments were repeated three times. #, undetected. ****, *P < *0.0001 (by an unpaired two-tailed *t* test with Welch’s *post hoc* correction [C and D]).

To further determine the receptor of E18, we carried out screening for host factors required for E18 infection using the CRISPR/Cas9 membrane protein knockout system constructed in rhabdomyosarcoma (RD) cells as we described in our previous work (24). The E18-HeB15-254 strain was used to infect the RD cell pool. At 3 days postinfection, the surviving cells were harvested, and genomic DNA was extracted. The single guide RNA (sgRNA)-coding regions were amplified from the original pool, and the surviving cells were then used for next-generation sequencing (NGS) (Fig. 2A). All the data were analyzed using the model-based analysis of genome-wide CRISPR/Cas9 knockout (MAGeCK) method; *FCGRT* and *B2M*, the two genes encoding the FcRn subunits, were identified as the essential genes for E18 infection (Fig. 2B; see also Table S1 in the supplemental material). Unlike E6, FcRn was identified as the only candidate for the E18 receptor.

10.1128/mbio.01166-22.5 TABLE S1 Identification of genes involved in echovirus 18 replication by the MAGeCK method. Download Table S1, XLSX file, 0.4 MB. Copyright © 2022 Chen et al. 2022 Chen et al. https://creativecommons.org/licenses/by/4.0/ This content is distributed under the terms of the Creative Commons Attribution 4.0 International license.

Next, we sought to determine whether the ectopic expression of *FCGRT* or *B2M* in the knockout cells restored their sensitivity to E18. The knockout cell lines were transduced with *FCGRT*- and *B2M*-expressing lentivirus for the ectopic expression of FcRn. The resulting cell lines restored susceptibility to E18 infection (Fig. 2C), suggesting that FcRn is critical for E18 infection.

To further confirm the role of FcRn as an essential receptor for E18, E18 infection in echovirus-nonpermissive Chinese hamster ovary (CHO) cells and the corresponding human FcRn cell lines was evaluated. The ectopic expression of human FcRn rendered CHO cells sensitive to E18 infection (Fig. 2D).

### E18 binds and interacts with the extracellular domain of FcRn directly.

**FIG 3:**
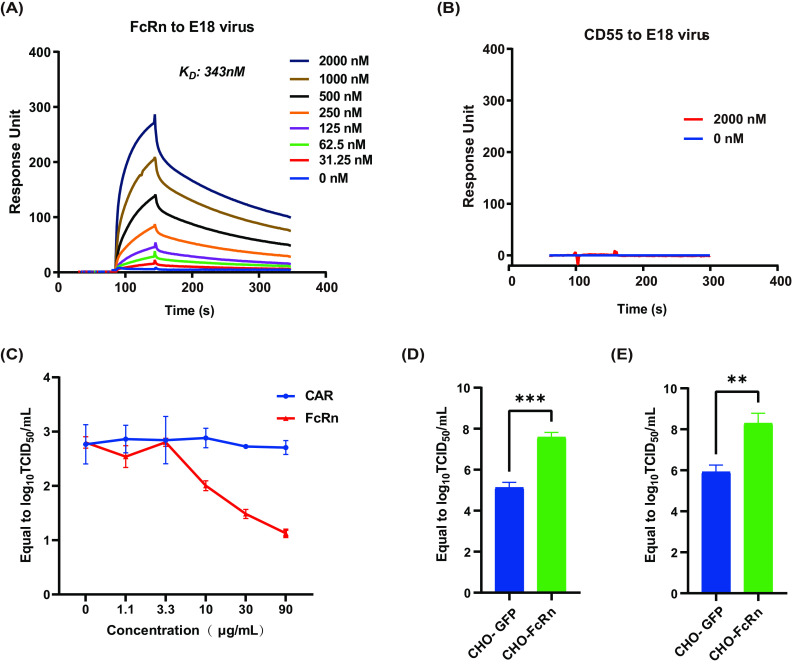
Direct binding between FcRn and E18 viral particles. (A and B) SPR assay characterizing the binding between E18 full particles and FcRn (A) or CD55 (B). The *K_D_* values were calculated using Biacore 3000 analysis software (BIAevaluation version 4.1). (C) Soluble FcRn inhibits E18 infection dose dependently. CAR was used as a control. (D and E) Binding (D) and endocytosis (E) of E18 in green fluorescent protein-expressing CHO-GFP or human FcRn-expressing CHO-FcRn cells. Data depict the means with SEM (*n* = 3). **, *P < *0.01; ***, *P < *0.001 (by an unpaired two-tailed *t* test with Welch’s *post hoc* correction).

To validate the direct interaction between E18 and FcRn, surface plasmon resonance (SPR) experiments were performed to examine the interaction between FcRn and E18 viral particles (Fig. 3; Fig. S1). The ectodomain of FcRn binds to E18 with high affinity (equilibrium dissociation constant [*K_D_*] = 343 nM) at pH 7.4 (Fig. 3A). CD55 serves as an attachment receptor for many picornaviruses (24), so the binding between CD55 and E18 was also evaluated. CD55 showed no binding affinity for E18 particles (Fig. 3B).

10.1128/mbio.01166-22.1 FIG S1 Expression and purification of the FcRn soluble protein. Shown are size exclusion chromatograms and SDS-PAGE analysis of FcRn expressed in 293T cell lines. The two plasmids encoding the ectodomains of FCGRT and B2M were cotransfected into 293T cells. Soluble FcRn was purified by nickel affinity chromatography and further characterized by using a Superdex 200 column (GE Healthcare). Download FIG S1, TIF file, 1.7 MB. Copyright © 2022 Chen et al. 2022 Chen et al. https://creativecommons.org/licenses/by/4.0/ This content is distributed under the terms of the Creative Commons Attribution 4.0 International license.

Once we confirmed the interaction between FcRn and purified E18 *in vitro*, we further determined the binding between E18 virions and FcRn. Soluble FcRn incubated with E18 virions in Dulbecco’s modified Eagle medium (DMEM) inhibited E18 infection in a dose-dependent manner, further supporting that FcRn is the receptor for E18 under physiological conditions (Fig. 3C).

To further verify whether E18 virions could directly bind to FcRn, CHO cells expressing green fluorescent protein (GFP) (CHO-GFP cells) and CHO cells expressing human FcRn (CHO-FcRn cells) were incubated with E18 viral particles. The results showed that FcRn had a significant effect on E18 attachment to the cell surface (Fig. 3D). Viral internalization was also increased in FcRn-expressing CHO cells (Fig. 3E). These results further confirmed that FcRn acts as the receptor for E18.

### Structural analysis of E18 with FcRn.

**FIG 4:**
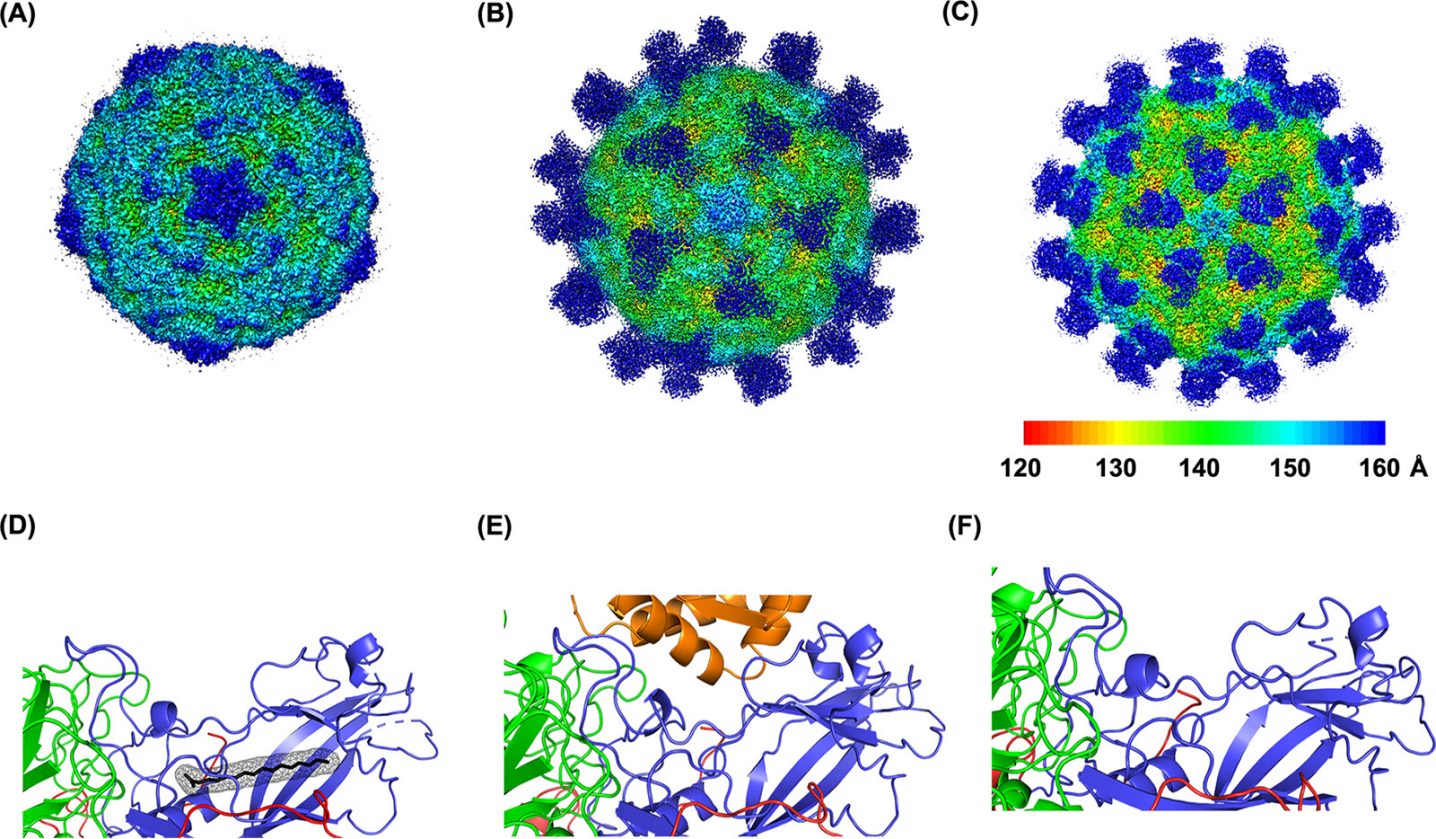
Cryo-EM structure of E18 and its complex with receptor FcRn. (A to C) Density maps of full E18 particles at pH 5.5 (A) and E18 in complex with FcRn at pH 7.4 (B) or pH 5.5 (C). (D) Closeup view of the hydrophobic pocket and the pocket factor inside. (E and F) Collapsed pocket without the pocket factor. The density maps are colored by radius, as shown in the key in panel C. Atomic models of the protein are shown in ribbons, and VP1, VP2, VP3, and FcRn heavy chains are shown in blue, green, red, and orange, respectively.

To better understand how FcRn mediates infection by E18, we characterized their interaction in detail using cryo-EM. E18 (strain HeB15-254) was purified by sucrose density centrifugation, and the fractions of mature virus particles were collected. Detailed characterizations of mature E18 particles (Fig. 4A and D), the E18/FcRn complex under physiological conditions (pH 7.4) (Fig. 4B and E), and the E18/FcRn complex under acidic conditions (pH 5.5) (Fig. 4C and F) were performed (Fig. 4). The density map of all three particles showed clear protein backbones and side chains of most residues, which allowed us to build the atomic model.

The E18 mature particle has a pseudo-T=3 symmetry arrangement resembling other picornavirus architectures. Protomers consist of the capsid protein (VP1, VP2, and VP3) and the smaller internal protein VP4. Sixty copies of the protomer-packaged mature particles had a diameter of 30 nm. E18, along with other enteroviruses, has a hydrophobic pocket within the hydrophobic β-barrel core of VP1, which occupies a natural fatty acid “pocket factor.”

The binding of FcRn to the cannon of the E18 particle displaces the pocket factor and then destabilizes the virus to initiate uncoating (Fig. 4A and B). A decrease in the pH further accelerates the release of the pocket factor (Fig. 4C). Accompanied by pocket factor release, FcRn dissociates from the virus particles, and the unstable virus particles initiate uncoating. The density of FcRn around the particles was observed and compared to that of the complex at pH 7.4.

### Molecular mechanism of E18 interaction with FcRn.

**FIG 5:**
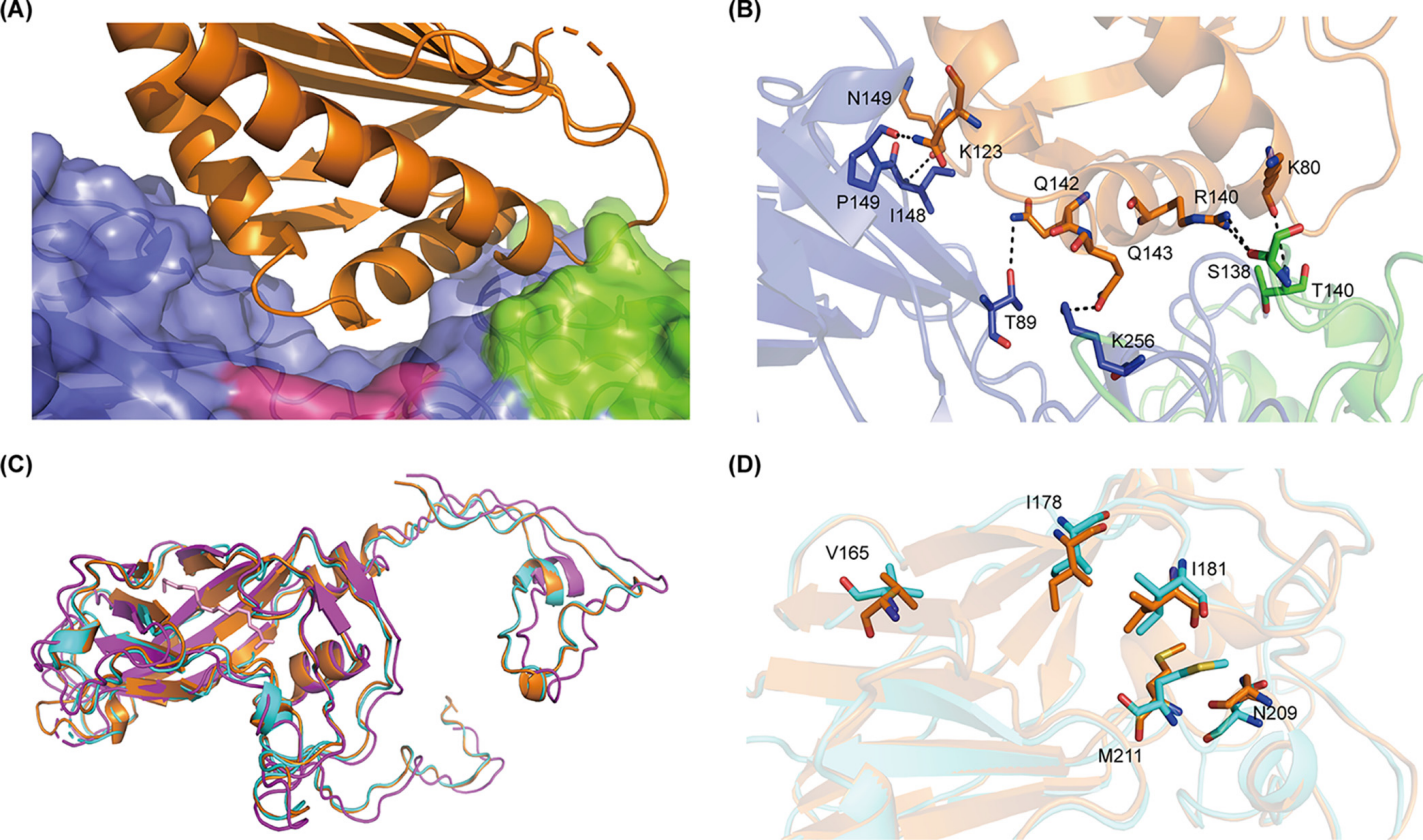
The molecular mechanism of the E18 interaction with FcRn. (A) Atomic model of the E18/FcRn complex in the main contacting interface. (B) Details of the interaction between E18 and FcRn. The proteins are colored by chain as shown in Fig. 4. (C) Alignment of VP1 protein from free E18 particles and the E18/FcRn complex at pH 7.4 or pH 5.5. (D) Residues that played a key role in pocket factor release and pocket collapse.

We found that FcRn binds to virions at its canyon site (Fig. 5A). VP1 and VP2 interact with domain 3 of FcRn. Specifically, residues T89, I148, P149, and K256 in VP1 form hydrogen bonds (H-bonds) with Q142, K123, N149, and Q143 in FcRn, respectively. S138 and T140 in VP2 form H-bonds with R140 and K80 in FcRn, respectively (Fig. 5B). The binding of FcRn had a minimal impact on the overall capsid structure. When the pH of the complex was reduced to pH 5.5, rotation of the residues around the hydrophobic pocket that play a key role in stabilizing the pocket factor was observed. The rotation of these residues further displaced the pocket factor from the pocket of VP1.

The phylogenetic tree showed that E18 could be divided into three genotypes, i.e., A, B, and C. Genotype C was further divided into subgenotypes C1 to -4, while only two subgenotypes were reported in previous studies (12). Almost all the E18 *VP1* sequences reported from China after 2015 clustered together and formed subgenotype C4, except for HeB15-54462 and LC/48/17/E18, which belong to subgenotype C3 and are clustered with sequences from the United States, France, Australia, the Netherlands, South Korea, and Thailand (Fig. S2). All binding sites that interacted with FcRn (T89, I148, P149, and K256) of protein VP1 and T140 of protein VP2 were conserved, while S138 of protein VP2 contained an S138N substitution in many prevalent strains worldwide (Fig. S3).

10.1128/mbio.01166-22.2 FIG S2 Phylogenetic analysis of E18 based on the *VP1* sequences. The sequences in red indicate E18 strains in China. The green points indicate the E18 strains used in this study. Genotype C is the prevalent genotype in recent years, while subgenotype C4 is formed by the sequences from China after 2015. Download FIG S2, EPS file, 3.3 MB. Copyright © 2022 Chen et al. 2022 Chen et al. https://creativecommons.org/licenses/by/4.0/ This content is distributed under the terms of the Creative Commons Attribution 4.0 International license.

10.1128/mbio.01166-22.3 FIG S3 Alignment of the key residues in VP1 and VP2 of E18 responsible for FcRn binding. The VP1 and VP2 protein sequences of representative E18 epidemic strains were aligned. The residues involved in the interaction with FcRn are colored pink. Download FIG S3, TIF file, 4.2 MB. Copyright © 2022 Chen et al. 2022 Chen et al. https://creativecommons.org/licenses/by/4.0/ This content is distributed under the terms of the Creative Commons Attribution 4.0 International license.

### FcRn-decorated liposomes facilitate E18 uncoating.

**FIG 6:**
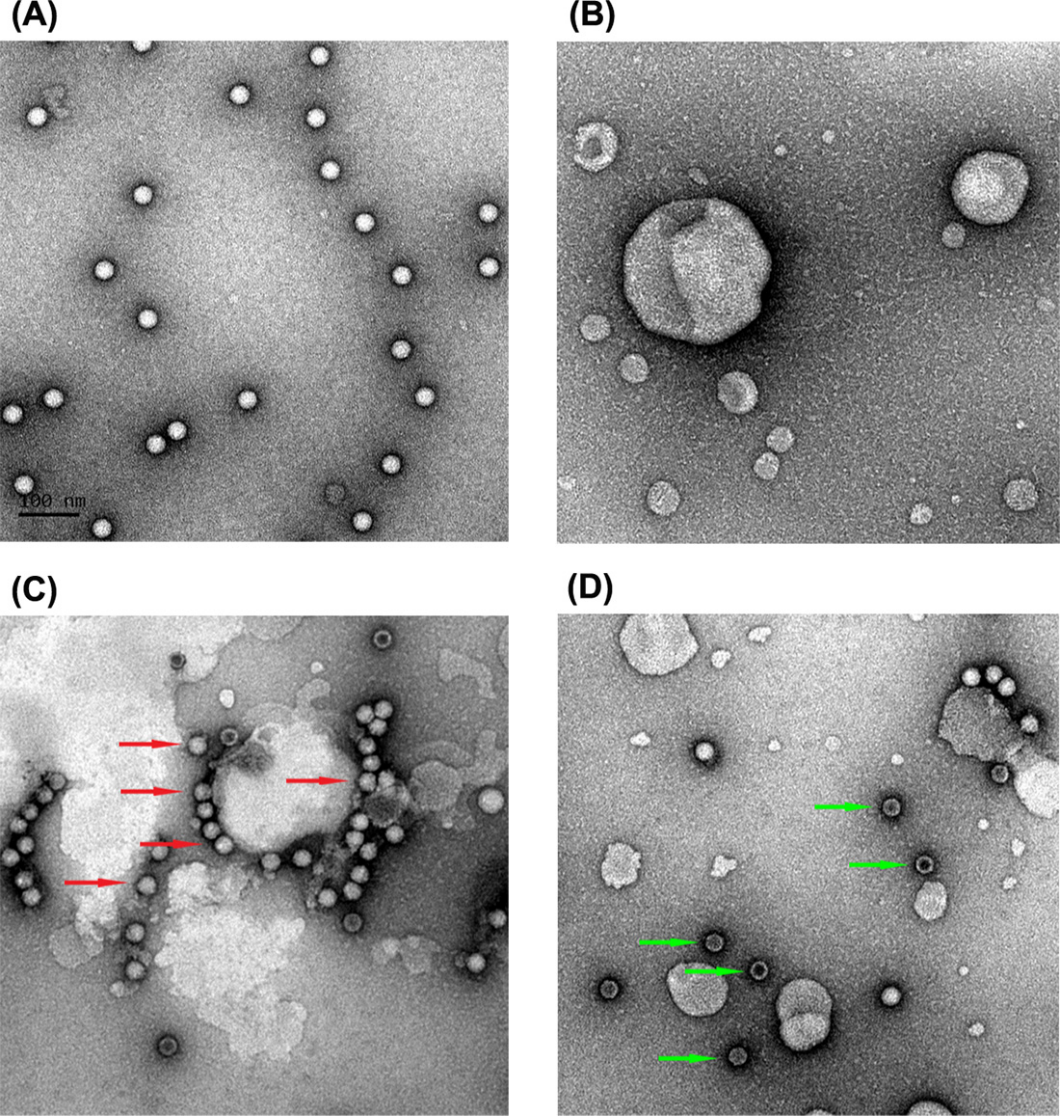
FcRn-decorated liposomes facilitate E18 uncoating. (A and B) Negative-staining EM of purified E18 (A) and nickel-charged liposomes (B). (C) E18 was incubated with FcRn-decorated liposomes at pH 7.4. Red arrows indicate the particles attached to the liposomes’ surface. (D) E18 was incubated with FcRn-decorated liposomes at pH 5.5. Green arrows indicate the particles released from the liposomes’ surface.

After E18 binds to the cell surface receptors, a small membrane region pinches around the virus to form an endosome. Interactions with the receptor protein under acidic conditions in the endosome trigger a rearrangement of capsid proteins, releasing the viral genome into the cytoplasm. To investigate whether soluble FcRn could induce E18 virion uncoating *in vitro*, E18 mature particles were incubated with FcRn under either neutral (pH 7.4) or acidic (pH 5.5) conditions at 37°C, and the virion morphologies were then compared by negative-stain electron microscopy. Consistent with the SPR results, mature E18 particles bound the FcRn-decorated liposomes under neutral pH, and no further conformational changes were observed (Fig. 6C). When incubating mature E18 particles with FcRn-decorated liposomes at acidic pH, the particles are first bound to the liposomes and then transformed into empty particles and released from the FcRn-decorated liposomes (Fig. 6D). The mature E18 particles alone did not exhibit significant changes under acidic conditions (Fig. 6).

## DISCUSSION

**FIG 7:**
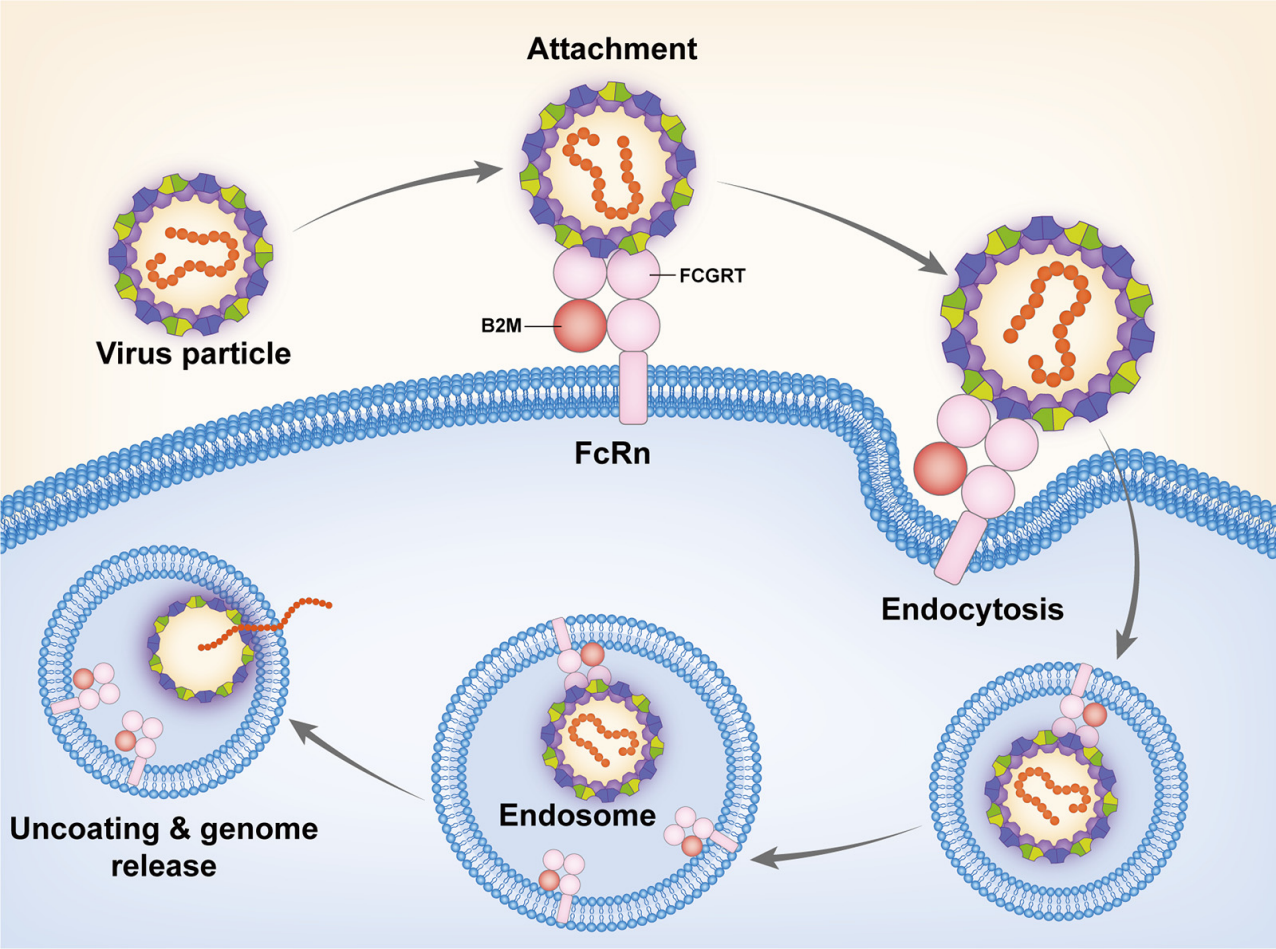
Model for the entry of certain echovirus serotypes into the host cell. The structural proteins VP1, VP2, VP3, and VP4 are shown in dark blue, dark green, light green, and purple, respectively. The viral RNA genome is indicated in orange. The FCGRT domain and B2M domain of FcRn are shown in pink and red, respectively.

Receptor usage determines viral tropism and its pathogenesis. Many picornaviruses use dual-receptor systems to infect cells (24–28). Attachment receptors work to facilitate the binding of viral particles to the cell surface; uncoating receptors mediate the uncoating of virus particles on the acidic endosome membrane. The binding of the attachment receptor does not trigger the release of the genome. However, the uncoating receptor itself triggers the rearrangement of capsid proteins, leading to the formation of a channel that connects the capsid and the endosomal membrane, and the viral genome is then released into the cytoplasm (29–31). We describe the mechanism for the entry of certain EV-B serotypes by using FcRn as a two-in-one attachment-and-uncoating receptor. For example, E18 binds to FcRn on the cell surface and enters the cells via endocytosis and internalization. FcRn further acts as an uncoating receptor in the endosomes, triggering virus uncoating and genome release (Fig. 7). There are also several other enteroviruses that use a single receptor for both steps. Poliovirus belongs to EV-C, which uses CD155 (Poliovirus Receptor, PVR) as a bifunctional receptor (32). KREMEN1 works as both an attachment and an uncoating receptor for Coxsackievirus A10 (CVA-10) (31). EV-Bs, such as CVB-3, use coxsackievirus and adenovirus receptor (CAR) as the uncoating receptor and selectively use CD55 as a binding receptor, determined by a single mutation on VP3-234 (28). These results indicated that a separate binding receptor is not essential for enterovirus entry.

CD55 functioned as the attachment receptor for many picornaviruses (24, 30, 33–39), but the mechanism of CD55 binding to different virus particles is not the same. According to our study, E18 did not bind to CD55 at all. Structure-based alignment of E18 to E6/CD55 complex showed that VP2 K140 in E6 forms H-bonds with S155 of CD55, while in E18, T140 of VP2 could not form H-bonds. In the E6/CD55 complex, VP2 T163 forms H-bonds with CD55 D240, and VP2 P142 forms H-bonds with CD55 S157; E18 contains the same T163 and P142 residues but does not form H-bonds with CD55. The different spatial orientations of VP1 Q199 and G201 and VP2 V154 are also abolished in the interaction with CD55 (see Fig. S4A in the supplemental material). Alignment of E18 to the E11/CD55 complex revealed that the substitutions in VP2 (T161, K163, and K164) and VP3 (K63) as well as the different spatial orientations of VP2 (S157 and S159) abolish the interaction between E18 and CD55 (Fig. S4B).

10.1128/mbio.01166-22.4 FIG S4 Alignment of E18 to the E6/CD55 complex (A) and the E11/CD55 complex (B). Residues that may be involved in the interaction with CD55 are shown as sticks. The residues of E6, E11, E18, and CD55 are shown in green, light blue, gray, and wheat, respectively. Download FIG S4, TIF file, 2.0 MB. Copyright © 2022 Chen et al. 2022 Chen et al. https://creativecommons.org/licenses/by/4.0/ This content is distributed under the terms of the Creative Commons Attribution 4.0 International license.

Fever, headache, convulsions, meningeal irritation symptoms, and disturbance of consciousness are the main clinical manifestations of E18 infection. Most patients have a favorable prognosis. However, serious infections can cause severe damage to the CNS and threaten patients’ lives, especially children. AM is the most commonly reported syndrome associated with E18 infection. However, acute transverse myelitis, VE, flaccid paralysis, viral sepsis, shock, and death have also been documented. E18 is also a common EV detected in infants (2, 40), and it most frequently causes neonatal sepsis (40). Severe neonatal sepsis is usually accompanied by multiple-organ dysfunction syndromes, with an 18.8% hospital mortality rate (41). FcRn is a significant immune factor for neonates, transporting maternal IgG antibodies from the gut to the bloodstream across the intestinal epithelium. This study demonstrates that this key immune factor is hijacked by E18 as a receptor for its invasion, and it has both binding and uncoating functions. The distribution of FcRn in the lungs, kidneys, gut, vascular endothelium, and blood-brain barrier corresponds to the tropism of E18 and its relevant clinical manifestations.

## MATERIALS AND METHODS

### Cells and viruses.

RD, 293T, and CHO cells were maintained in Dulbecco’s modified Eagle medium (DMEM; Invitrogen) supplemented with 10% fetal bovine serum (FBS; Invitrogen) and 1% penicillin-streptomycin. All these cells were cultured at 37°C with 5% CO_2_.

Viruses in this study included E18 (HeB15-254 and WF277), E2 (XJ2011-2922001), and E15 (CH 96-51). RD cells were used to propagate these viruses.

### Protein purification.

The soluble ectodomains of FcRn and CD55 were prepared as described previously (24). Briefly, the constructs containing the coding fragments of human Fc fragment of IgG receptor and transporter (*FCGRT*) (A16-L282) and *B2M* were cotransfected into 293T cells for the expression of FcRn, and constructs containing the coding fragment of human CD55 (D35-G285) were transfected into 293T cells to express CD55. All the genes used here were synthesized by Genewiz and inserted into a pCMV3 vector (Sino Biological, China). After the vector was propagated and centrifuged, the supernatants were further purified by Ni-nitrilotriacetic acid (NTA) chromatography and then by Hiload 16/600 Superdex 200 PG column (GE Healthcare) gel chromatography with a buffer containing 20 mM Tris-HCl and 150 mM NaCl (pH 8.0).

### CRISPR/Cas9 screening.

RD-Cas9 sgRNA libraries were infected with E18 (strain HeB15-254) at a multiplicity of infection (MOI) of 0.01. After 48 h, the medium was removed, and the cells were washed intensively to remove dead cells. The remaining cells were collected, and the genomic DNA was extracted. The sgRNA-coding regions within the genomic DNA from both untreated cell libraries (two biological replicates) and experimental groups (two biological replicates) were amplified by PCR (primers CaslibF [TATCTTGTGGAAAGGACGAAACACC] and CaslibR [AATACGGTTATCCACGCGGC]) and identified by deep sequencing analysis. Data analyses were conducted using the previously reported MAGeCK method (42).

### Viral TCID_50_ analysis on KO and *trans*-complemented cell lines.

Serially diluted viruses (from 10^−1^ to 10^−10^) were added to 293TWT, KO (*FCGRT*^KO^, *B2M*^KO^, and *CD55*^KO^) and *trans*-complemented (293T *FCGRT*^KO^+Lenti-FCGRT and 293T *B2M*^KO^+Lenti-B2M) cells (24). CPE was observed after 96 h, and the 50% tissue culture infective dose (TCID_50_) was calculated according to the Reed-Muench method.

### Virus infection in nonpermissive CHO cells with *FCGRT* and *B2M* ectopic expression.

CHO cells were transduced with Lenti-FCGRT+Lenti-B2M or Lenti-GFP (24). E18 (MOI = 0.1) was inoculated into the cells at 37°C for 1 h and then removed, and the cells were washed twice with phosphate-buffered saline (PBS). After 72 h, the supernatant was collected for TCID_50_ analysis. TCID_50_ detection was performed in RD cells.

### Bright-field imaging of CPE.

For bright-field images, 293T WT, *FCGRT*^KO^, *B2M*^KO^, and *CD55*^KO^ cells were infected with E18 at an MOI of 1. Cells were imaged at 36 h postinfection, and CPE was read.

### Phylogenetic and amino acid variation analyses.

Nucleotide sequence alignment of the *VP1* gene of EV-B was performed using the MEGA program (version 6.0). Phylogenetic trees based on E18 entire *VP1* gene sequences were constructed using the neighbor-joining method implemented in the MEGA program with the Kimura 2-parameter model. Regions containing alignment gaps were excluded from the analysis. The robustness of the phylogenetic tree was tested using the bootstrap method with 1,000 replications. Amino acid substitution analysis was conducted using BioEdit software (version 7.2.5).

### Virus production and purification.

E18 was passaged in the RD cell line at an MOI of 0.005, cultivated at 37°C, and harvested at 60 h postinfection. The supernatant was centrifuged at 12,000 × *g* for 60 min to remove the cells and debris. A KrosFlo KR2i TFF system with a cutoff of 300 kDa was used to concentrate the supernatant. The concentrated products were loaded onto a continuous 20 to 50% sucrose density gradient and centrifuged at 120,000 × *g* at 4°C for 4 h. After buffer exchange, the viral particles were concentrated using an Amicon Ultra-6 100-kDa-cutoff centrifugal concentrator (Millipore).

### Surface plasmon resonance assay.

A Biacore 3000 spectrometer was used to characterize the binding kinetics between full E18 particles and FcRn or CD55 at 25°C. Biotinylated E18 in PBS with Tween was immobilized on streptavidin (SA) chips and analyzed for real-time binding by flowing-through FcRn or CD55 protein at concentrations ranging from 31.25 nM to 2,000 nM. Sensorgrams were analyzed using Biacore 3000 Evaluation software (version 1.5.0.0). *K_D_* values were calculated using a model of 1:1 binding.

### Binding and internalization assays.

Binding and internalization assays were performed as previously described, with slight modifications (24). CHO-FcRn and CHO-GFP cells were transduced with E18 (MOI = 50). Quantitative PCR (qPCR) was used to detect E18 (primers PEVF [TTGTCACCATWAGCAGYCA] and PEVR [CCTGAATGCGGCTAATCC] and probe PEV Probe [6-carboxyfluorescein {FAM}-CCGACTACTTTGGGWGTCCGTGT-6-carboxytetramethylrhodamine {TAMRA}]). Glyceraldehyde-3-phosphate dehydrogenase (GAPDH) was included as an internal control (qPCR primers HGapdhF [CCACTCCTCCACCTTTGAC] and HGapdhR [ACCCTGTTGCTGTAGCCA] and probe HGapdhProbe [FAM-TTGCCCTCAACGACCACTTTGTC-TAMRA]).

### Blocking assays with soluble FcRn.

RD cells were seeded into 96-well plates. Serial dilutions of purified FcRn or coxsackievirus and adenovirus receptor (CAR) soluble proteins were incubated with E18 (MOI = 0.1) for 2 h at 4°C in a volume of 100 μL. The mixture was added to RD cells, incubated at 37°C for 1 h, and then removed, and the RD cells were washed twice with PBS. DMEM with 2% FBS was added to the cells. After 17 h, the supernatants were collected for E18 detection by qPCR.

### Virus production and purification.

E18 was incubated in the RD cell line at an MOI of 0.01. Viruses were harvested 72 h after infection. The supernatant was clarified at 1,500 × *g* at 4°C for 1 h. The clarified supernatant was further concentrated using the KrosFlo KR2i TFF system with a cutoff of 300 kDa. The concentrated virus was loaded onto a continuous 20 to 50% sucrose density gradient and centrifuged at 120,000 × *g* for 4 h. Two bands corresponding to the empty and full particles were collected and dialyzed against PBS. The fractions were further validated using a negative-staining transmission electron microscope (JEOL-1400).

### Cryo-EM sample preparation, data collection, and processing.

For full-particle cryo-grid preparation, 3 μL of the purified virus in PBS was deposited onto freshly glow-discharged 300-mesh ultrathin C film on Lacey carbon grids. After a 60-s incubation at 4°C with 100% humidity, grids were blotted for 3 s for plunge-freezing (Vitro bot IV; FEI). To prepare the virus/receptor complex cryo-grids, the virus was mixed with FcRn at 4°C, at final concentrations of 3 mg/mL and 0.5 mg/mL, respectively. After incubation on ice, 3 μL of the virus/receptor complex was deposited onto grids; after a 60-s incubation, grids were blotted for 3 s for plunge-freezing. Data were collected using a Titan Kros microscope (FEI) with a K2 detector at a magnification of ×59,000 (pixel size of 1.41 Å) and fractionated into 39 frames with an accumulated dose of 40 e^−^/Å^2^, with a defocus range from −1 to −2 for image processing.

Movie frames were aligned and averaged using Motioncor2. The contrast transfer function parameters were determined using CTFFIND4. Particle picking was performed based on a Laplacian-of-Gaussian filter. The structures were calculated using Relion3 with an applied icosahedral symmetry. The rigid body was fitted onto the E18 map using UCSF’s E18 model (PDB accession number 6HBG) as a template. After that, the atomic model was manually adjusted by using Coot. The resulting coordinates were then used for further refinement using PHENIX. The quality of the final atomic model was evaluated using MolProbity.

### Statistical analysis.

Data were analyzed using GraphPad Prism software (version 7.00; GraphPad, La Jolla, CA, USA) by unpaired two-tailed *t* tests with Welch’s *post hoc* correction. Comparisons between the two groups were evaluated using the independent-sample *t* test. Comparisons of three or more groups were performed using one-way analysis of variance (ANOVA). These results are expressed as means ± standard deviations (SD). A *P* value of <0.05 was considered a statistically significant difference between values.

### Data availability.

The cryo-EM density maps of icosahedral reconstructions for E18/FcRn complex at pH 7.4, E18/FcRn complex at pH 5.5, and the mature E18 virus at pH 5.5 have been deposited in the Electron Microscopy Data Bank (https://www.ebi.ac.uk/pdbe/emdb/) under accession codes EMD-33499, EMD-33505, and EMD-33502, respectively. The corresponding atomic coordinates have been submitted to the Protein Data Bank with accession numbers 7XXA, 7XXJ, and 7XXG, respectively.
