# Supplementary figures and images for: Human FcRn Is a Two-in-One Attachment-Uncoating Receptor for Echovirus 18
Source: mBio. 2022 Jul 5;13(4):e01166-22. doi: 10.1128/mbio.01166-22 (PMC9426509; doi:10.1128/mbio.01166-22)

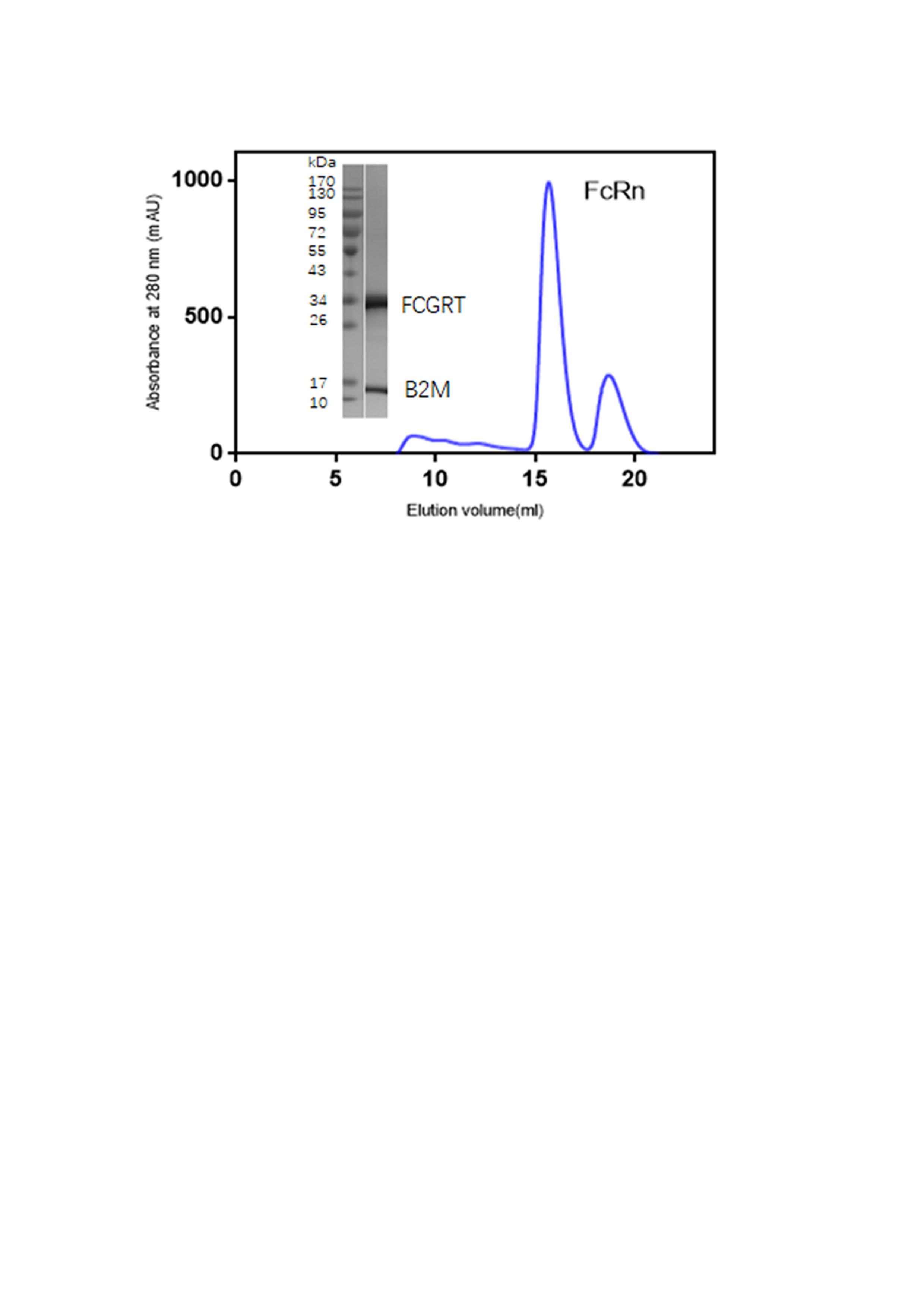

Supplement: FIG S1 [file mbio.01166-22-s0001.tif]

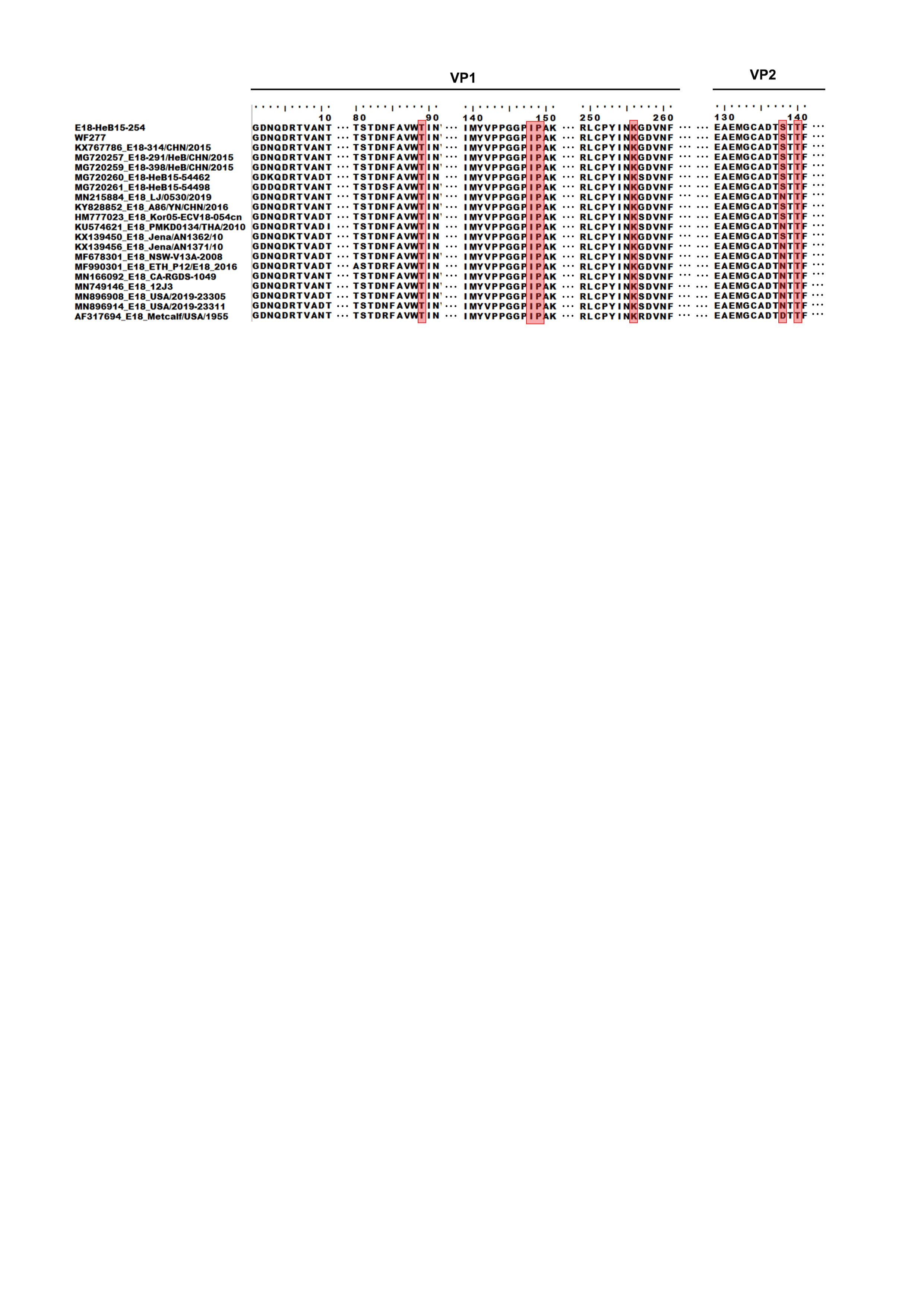

Supplement: FIG S3 [file mbio.01166-22-s0003.tif]

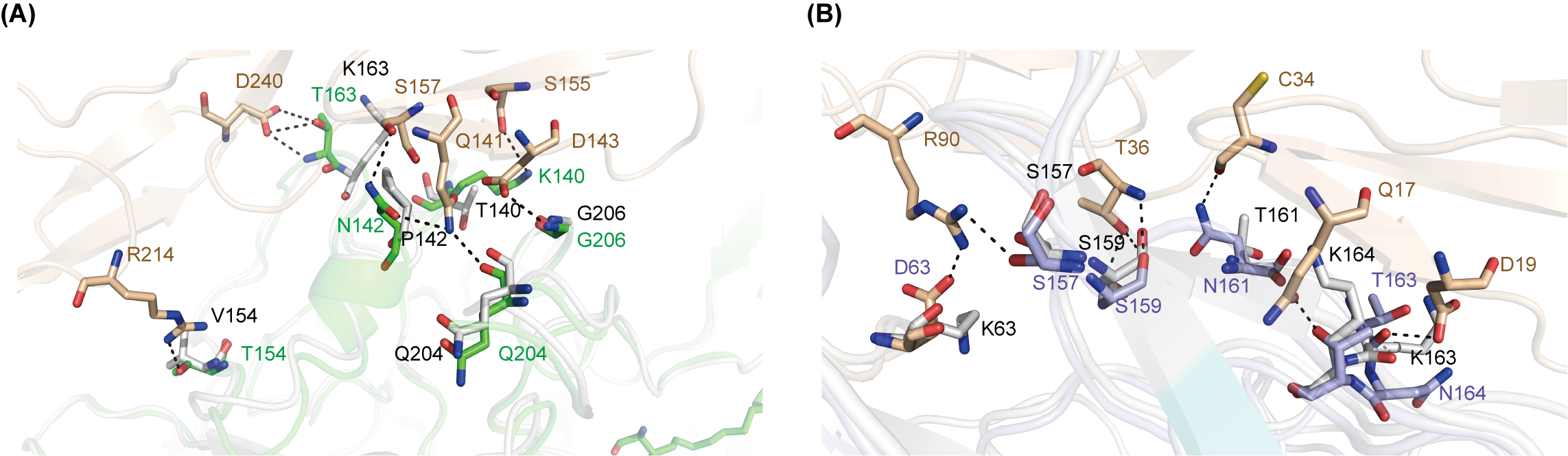

Supplement: FIG S4 [file mbio.01166-22-s0004.tif]
